# Dual MET–EGFR combinatorial inhibition against T790M-EGFR-mediated erlotinib-resistant lung cancer

**DOI:** 10.1038/sj.bjc.6604559

**Published:** 2008-08-26

**Authors:** Z Tang, R Du, S Jiang, C Wu, D S Barkauskas, J Richey, J Molter, M Lam, C Flask, S Gerson, A Dowlati, L Liu, Z Lee, B Halmos, Y Wang, J A Kern, P C Ma

**Affiliations:** 1 Division of Hematology/Oncology, Department of Medicine, Case Western Reserve University, University Hospitals Case Medical Center, Cleveland, OH, USA; 2 Department of Radiology, Case Western Reserve University, University Hospitals Case Medical Center, Cleveland, OH, USA; 3 Case Center for Imaging Research, University Hospitals Case Medical Center, Cleveland, OH, USA; 4 Case Comprehensive Cancer Center, Cleveland, OH, USA; 5 Division of Pulmonary, Critical Care and Sleep Medicine, Department of Medicine, Case Western Reserve University, University Hospitals Case Medical Center, Cleveland, OH, USA

**Keywords:** MET, EGFR, inhibitor, erlotinib, resistance, lung cancer

## Abstract

Despite clinical approval of erlotinib, most advanced lung cancer patients are primary non-responders. Initial responders invariably develop secondary resistance, which can be accounted for by T790M-*EGFR* mutation in half of the relapses. We show that MET is highly expressed in lung cancer, often concomitantly with epidermal growth factor receptor (EGFR), including H1975 cell line. The erlotinib-resistant lung cancer cell line H1975, which expresses L858R/T790M-EGFR in-*cis*, was used to test for the effect of MET inhibition using the small molecule inhibitor SU11274. H1975 cells express wild-type *MET*, without genomic amplification (CNV=1.1). At 2 *μ*M, SU11274 had significant *in vitro* pro-apoptotic effect in H1975 cells, 3.9-fold (*P*=0.0015) higher than erlotinib, but had no effect on the MET and EGFR-negative H520 cells. *In vivo*, SU11274 also induced significant tumour cytoreduction in H1975 murine xenografts in our bioluminescence molecular imaging assay. Using small-animal microPET/MRI, SU11274 treatment was found to induce an early tumour metabolic response in H1975 tumour xenografts. MET and EGFR pathways were found to exhibit collaborative signalling with receptor cross-activation, which had different patterns between wild type (A549) and L858R/T790M-EGFR (H1975). SU11274 plus erlotinib/CL-387,785 potentiated MET inhibition of downstream cell proliferative survival signalling. Knockdown studies in H1975 cells using siRNA against *MET* alone, EGFR alone, or both, confirmed the enhanced downstream inhibition with dual MET–EGFR signal path inhibition. Finally, in our time-lapse video-microscopy and *in vivo* multimodal molecular imaging studies, dual SU11274-erlotinib concurrent treatment effectively inhibited H1975 cells with enhanced abrogation of cytoskeletal functions and complete regression of the xenograft growth. Together, our results suggest that MET-based targeted inhibition using small-molecule MET inhibitor can be a potential treatment strategy for T790M-EGFR-mediated erlotinib-resistant non-small-cell lung cancer. Furthermore, optimised inhibition may be further achieved with MET inhibition in combination with erlotinib or an irreversible EGFR-TKI.

Receptor tyrosine kinases (RTKs) play a key role in lung cancer tumorigenesis and progression (Choong *et al*, 2005). Progress has been made in the treatment of advanced non-small-cell lung cancer (NSCLC) using small-molecule tyrosine kinase inhibitors (TKIs) gefitinib and erlotinib, targeting epidermal growth factor receptor (EGFR) (Lynch *et al*, 2006). *EGFR* kinase domain mutations (frequently L858R) and exon 19 deletions have been identified to be predictive of response to gefitinib/erlotinib (Shigematsu and Gazdar, 2006; Sharma *et al*, 2007). Although erlotinib was shown to prolong survival in a large phase III randomised trial (NCIC-BR.21) (Shepherd *et al*, 2005), the majority of unselected lung cancer patients are still primary non-responders. Patients whose cancer has wild-type *EGFR* genotype are generally non-responders but may at best derive stable disease from the TKIs. Initial responders with mutant *EGFR* invariably develop secondary resistance and soon succumb to the disease. At least half of the acquired resistance is mediated by the ‘gatekeeper’ mutation T790M-*EGFR* (Kobayashi *et al*, 2005a; Pao *et al*, 2005). Moreover, T790M was found in the H1975 cell line, in combination with L858R, which was previously established without prior exposure to EGFR TKIs. Hence, T790M may also have a role in primary EGFR-TKI resistance. Currently, there are still no Federal Drug Administration (FDA)-approved clinical inhibitors that can overcome T790M-mediated EGFR-TKI resistance yet.

The MET receptor has been shown to be an important molecule in a variety of malignancies (Tsarfaty *et al*, 1994; Schmidt *et al*, 1997, 1998, 1999; Birchmeier *et al*, 2003; Ma *et al*, 2003b; Benvenuti and Comoglio, 2007), and has recently been validated as an attractive therapeutic target in cancer therapy, including lung cancer (Ma *et al*, 2003a, 2005a, 2005b; Christensen *et al*, 2005; Salgia, 2006). Overexpression of MET or its ligand HGF have been found to confer a poor prognosis (Ma *et al*, 2003b; Miyata *et al*, 2006; Garcia *et al*, 2007; Sawada *et al*, 2007). Dysregulation of the MET–HGF signalling axis upregulates diverse tumour cell functions, including cell proliferation, survival, cell scattering and motility, epithelial-mesenchymal transition, angiogenesis, invasion, and metastasis (Corso *et al*, 2005; Dietrich *et al*, 2005; Ma *et al*, 2005a; Peruzzi and Bottaro, 2006). Reversible small-molecule inhibitors such as SU11274 targeting MET have been developed for therapeutic inhibition (Christensen *et al*, 2003; Sattler *et al*, 2003; Ma *et al*, 2005a, 2005b). We hypothesised that MET signalling plays a key role in lung cancer oncogenic signalling and optimised therapy targeting MET would be effective as a treatment strategy in the face of EGFR-TKI resistance. In this study, we sought to define the role of MET signalling in EGFR-TKI-resistant lung cancer. Furthermore, using a combination of small-molecule kinase inhibitors and short-interfering RNA (siRNA), we examined the role of MET inhibition, either alone or combined with EGFR inhibition, using both *in vitro* and *in vivo* assays against the EGFR-TKI-resistant lung cancer cell line H1975 (L858R/T790M-mutant EGFR). Our data support the potential role of dual TKI combinatorial inhibition using EGFR inhibitors to enhance MET inhibition in T790M-EGFR-mediated therapy resistance.

## Materials and methods

### Cell culture, cell lysates preparation, immunoprecipitation, and immunoblotting

Lung cancer cell lines were obtained from American Type Culture Collection and grown in RPMI 1640 (Hyclone, Logan, UT, USA), 10% (v/v) foetal bovine serum (FBS) as instructed under standard cell culture conditions. For growth factor stimulation studies, human HGF (50 ng ml^−1^) (R&D Systems, Minneapolis, MN, USA) and human EGF (100 ng ml^−1^) (Calbiochem, Cambridge, MA, USA) were used as indicated. Cellular proteins were extracted from whole cells as previously described (Choong *et al*, 2006). Immunoprecipitation (IP) studies and immunoblotting (WB) were performed as previously described (Maulik *et al*, 2002; Choong *et al*, 2006) using the following primary antibodies as indicated: p-MET[Y1234/1235] (i.e., pY1252/1253 as in the full-length MET version) (Cell Signaling, Danvers, MA, USA), MET (C-12, Santa Cruz Biotechnology, Santa Cruz, CA, USA), p-EGFR[Y1068] (Cell Signaling), EGFR (Santa Cruz Biotechnology), p-ERBB3[Y1289] (Cell Signaling), p-AKT[S473] (Cell Signaling), AKT (Biosource-Invitrogen, Carlsbad, CA, USA), p-extracellular signal-regulated kinases 1 and 2 (ERK1/2)[T202/Y204] (Cell Signaling), ERK1/2 (Biosource-Invitrogen), p-STAT3[Y705] (Cell Signaling), STAT3 (Zymed-Invitrogen, Carlsbad, CA, USA), phosphotyrosine (p-Tyr; Upstate-Millipore, Billerica, MA, USA), and Actin (Santa Cruz Biotechnology).

### Chemicals

*SU11274*: [(3Z)-*N*-(3-chlorophenyl)-3-({3,5-dimethyl-4-[(4-methylpiperazin-1-yl)carbonyl]-1*H*-pyrrol-2-yl}methylene)-*N*-methyl-2-oxo-2,3-dihydro-1*H*-indole-5-sulphonamide] (Ma *et al*, 2005a) and CL-387,785 were purchased from EMD-Calbiochem (Cambridge, MA, USA), dissolved in DMSO, and used at the indicated concentrations. Erlotinib was prepared as previously described (Choong *et al*, 2006).

*Genomic studies of* MET *gene DNA extraction and DNA sequencing*: Genomic DNA of H1975 cells were extracted using Qiagen DNAeasy Kit (Qiagen, Valencia, CA, USA) according to the manufacturer's instructions. Direct DNA sequencing of the complete *MET* gene was performed as previously described (Ma *et al*, 2005a).

*Quantitative real-time polymerase chain reaction (QPCR)*: Genomic copy number variation of the *MET* gene was determined in triplicate using QPCR with the RNaseP as the reference gene. Quantitative real-time polymerase chain reactions were performed in ABI PRISM 7900-HT System and the reaction conditions are available upon request. The QPCR primers for *MET* copy number variation determination were purchased from ABI (ABI assay no.: Hs01565582_g1).

*Cellular apoptosis and viability assay*: For cellular apoptosis assays, cells were plated separately in triplicate in six-well plates in 10% FBS-containing media. Drug inhibitor treatment using erlotinib, SU11274, or DMSO control as indicated was added the next day, with the cells incubated for 72 h further. Cellular apoptosis was determined by fluorescence-activated cell sorting (FACS) analysis using the Annexin-V-Fluos Staining Kit (Roche Diagnostics, Mannheim, Germany) according to the manufacturer's instructions. Cellular apoptosis assays were performed in triplicate. Cellular viability assays were performed using the Trypan-Blue Dye Exclusion assay with duplicate counting using standard haemacytometer-light microscopy, with each experiment further repeated in duplicate.

### Time-lapsed video microscopy and image analysis of cytoskeletal functions

H1975 cells were plated on cell culture dishes and placed into a temperature-controlled chamber at 37°C in an atmosphere of 5% CO_2_. The cells were examined by and recorded under video microscopy using an Leica 6000 B inverted microscope, Pecon incubation chamber, and Retiga EXI 12 bit camera (Q imaging, Vancouver, BC, Canada) with MetaMorph image analysis software (Universal Imaging, Downington, PA, USA) (details see also Supplementary Materials and Methods).

*Lentivirus transduction of luciferase-expressing vector* (a) Lentivirus production: *Plasmids*. The packaging plasmid pCMVÄR8.91, the vesicular stomatitis virus glycoprotein G (VSV.G), and encoding plasmid pCSO-rre-cppt-MCU3-LUC were kind gifts from Dr Donald B Kohn (University of Southern California). *Virus production.* Transfection with transfer vector, packaging plasmid and envelope plasmid were performed by calcium phosphate precipitate 12 h after planting package 293T cells into 10 cm cell culture dishes. (b) Lentiviral transduction of EGFR-TKI-resistant lung tumour cells: Medium from the package cell culture was then collected and centrifuged at 3000 r.p.m. for 5 min at room temperature, followed by filtering through 0.45 *μ*m filter. The filtered medium containing virus particles was then added to the target transduction cells (H1975), which were plated the day before transduction.

### *In vivo* murine xenograft model

Six-week-old female Ncr-nu (Nude) mice were purchased from Charles River Laboratories (Wilmington, MA, USA) and hosted in the pathogen-free animal facility at the Case Western Reserve University. *In vivo* animal studies were performed according to institution-approved protocols and guidelines. Xenografts of the luciferase-expressing H1975 lung cancer cells were established by intradermally injecting 3 × 10^6^ viable cells in RPMI 1640 media into the flank/leg region of nude mice to produce subcutaneous tumours. Indicated treatments with targeted TKIs were given at the time when tumour xenografts were beginning to be visible (corresponding to 7 days post-implantation of H1975 cells). *In vivo* daily inhibitor drug treatments were performed as indicated. SU11274 was administered as intratumoral injections, whereas erlotinib was administered using oral gavage. Body weight was recorded for each animal twice weekly to monitor potential toxicities. Tumour xenografts were subsequently dissected and harvested at the end of the experiments, formalin-fixed, and stained with haematoxylin and eosin (H&E) using standard techniques.

*Small animal* in vivo *imaging* (a) Bioluminescence imaging (BLI): Xenograft tumour growth of H1975-luc cells were monitored by non-invasive luciferase-bioluminescence molecular imaging. Mice were imaged by BLI using a Xenogen IVIS® 200 bioluminescence scanner (Xenogen, Hopkinton, MA, USA) at indicated times on the pretreatment day as baseline, and then on various post-TKI treatment days as specified (details see also Supplementary Materials and Methods). (b) MicroPET/magnetic resonance imaging (MRI) imaging: For microPET/MRI imaging study, H1975 tumour xenografts were allowed to grow to a readily visible size in a total of 7 days post-implantation to ensure adequate baseline micro-PET uptake. H1975 tumour xenografts were treated with (a) diluent control and (b) SU11274 (100 *μ*g per xenograft). The nude mice were subjected to MRI (Bruker Biospec 7T MRI scanner, Bruker BioSpin MRI, Billerica, MA, USA) and microPET scanning (R4 micro-PET system, Siemens Medical Solutions, Knoxville, TN, USA) at the indicated time of treatment (details see also Supplementary Materials and Methods).

### siRNA inhibition

Specific siRNAs targeting human *MET* or *EGFR* mRNA, ON-TARGET plus SMARTpool, were purchased from Dharmacon Inc. (Chicago, IL, USA). The siRNA duplexes were transiently transfected using DharmaFECT 1 Transfection reagent (Dharmacon Inc.) according to the manufacturer's instructions. Control transfection using scrambled siRNA was performed in parallel using ON-TARGETplus siCONTROL siRNA (Dharmacon Inc.).

### Statistical analysis

Statistical significance was tested by two-tailed Student's *t*-test, with *P*-value of less than 0.05 considered statistically significant.

## Results

### Co-expression of MET and EGFR in lung cancer

**Figure 1:**
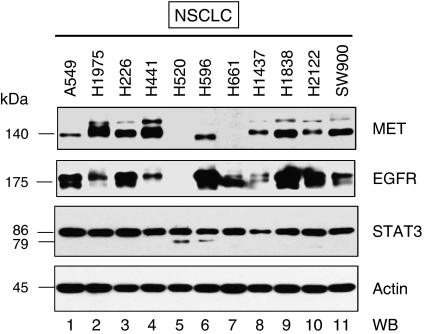
Co-expression pattern of MET and EGFR in lung cancer. The expression pattern of MET (1st panel) and EGFR (2nd panel) was examined using standard immunoblotting of the whole cell lysates (WCLs) from the following lung cancer cell lines cultured under serum-containing conditions (10% FBS): A549, H1975, H226, H441, H520, H596, H661, H1437, H1838, H2122, and SW900. The downstream signalling effector STAT3 (3rd panel) was also included in the immunoblot analysis. *β*-Actin was included as loading control (bottom panel).

We first examined the expression pattern of MET and EGFR in lung cancer cell lines. Nine of 11 NSCLC cell lines (82%) (except H520 and H661) (see Supplementary Table 1 for baseline characteristics of the cell lines) co-expressed both MET and EGFR, including the H1975 cell line (Figure 1). Signal transducer and activator of transcription 3 (STAT3) is a common downstream signalling target for both MET and EGFR, and has been shown to be crucial in mediating the oncogenic potential of mutant EGFR signalling (Song *et al*, 2003). STAT3 was ubiquitously expressed in all the cell lines examined.

### SU11274 induces apoptosis and inhibition of cytoskeletal functions in erlotinib-resistant H1975 lung cancer cells expressing L858R/T790M-EGFR

**Figure 2:**
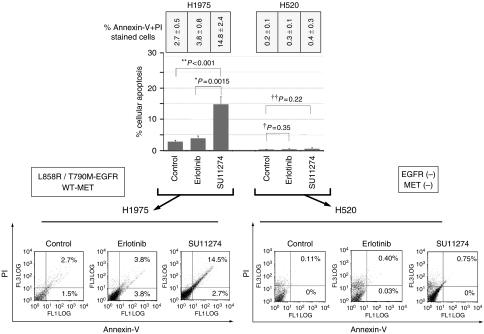
MET inhibition with SU11274 in EGFR-TKI-resistant H1975 lung cancer cells: induction of apoptosis *in vitro* and inhibition of cytoskeletal functions. The MET kinase inhibitor SU11274 was used to treat H1975 cells (L858R/T790M-*EGFR*, wild-type *KRAS*) and H520 cells (negative expression for both *EGFR* and *MET*) as control. The effect of SU11274 was examined using Annexin-V/propidium iodide (PI)-FITC cellular apoptosis assay. Untreated diluent control (U) and erlotinib were included in the experiment as treatment controls for comparison. Erlotinib (EGFR inhibition) was ineffective in promoting apoptosis in any of these above cell lines at 72 h. On the other hand, MET inhibition by SU11274 at 2 *μ*M induced significant cellular apoptosis in the EGFR-TKI-resistant H1975 cells (14.8±2.4%, *P*=0.0015), when compared with erlotinib (3.8±0.7%). For the EGFR-negative and MET-negative H520 cells, neither SU11274 (0.44±0.30%, *P*=0.22) nor erlotinib (0.28±0.13%, *P*=0.35) at 5 *μ*M induced any significant cellular apoptosis when compared with diluent control (0.18±0.10%). Mean values of percent cells in early apoptosis (Annexin-V plus PI staining) from three independent experiments for each of the treatment conditions were plotted in the graphs shown here. Error bar, s.e.m. (*N*=3).

As we found that many NSCLC cell lines co-express EGFR and MET, including H1975 cells, we first investigated if MET inhibition using the small-molecule inhibitor SU11274 can be effective in the erlotinib-resistant H1975 cells. H1975 cell line was chosen because it expresses the ‘gatekeeper’-resistant T790M-*EGFR* mutation (in-*cis* with L858R) in the receptor kinase domain hydrophobic pocket, representing a major mechanism of resistance to reversible EGFR-TKI (erlotinib/gefitinib) (Kobayashi *et al*, 2005a; Pao *et al*, 2005). SU11274 was previously characterised as a reversible inhibitor of MET, inhibiting specific tyrosine phosphorylation of the juxtamembrane CBL-binding phosphosite (pY1003), the major kinase autophosphorylation sites (pY1234/1235), as well as downstream signalling (Sattler *et al*, 2003; Ma *et al*, 2005a). It exhibits >60-fold selectivity for MET over FLK and >400-fold selectivity over RON, FGFR-1, SRC, CDK2, PDGFR-*β*, EGFR, and Tie-2 (Ma *et al*, 2005a). Here, we tested the pro-apoptotic effect of SU11274 treatment, in comparison to erlotinib, in the EGFR-TKI-resistant cell line H1975 (L858R/T790M-*EGFR*, wild-type *KRAS*). SU11274 at 2 *μ*M induced apoptosis (Annexin V- and propidium iodide-stained positive cells) in 14.8±2.4% of T790M-EGFR expressing H1975 cells, which is 5.5-fold (*P*<0.001) higher than diluent control and 3.9-fold (*P*=0.0015) higher than erlotinib. To further demonstrate that the pro-apoptotic effect of SU11274 seen above was not a result of off-target effects, we also tested the EGFR-negative and MET-negative H520 cell line as negative control. SU11274 at 5 *μ*M did not result in any significant apoptosis in H520 cells (0.44±0.30%, *P*=0.22). Similarly, H520 cells were also insensitive to erlotinib without any significant apoptosis induced by the drug (0.3±0.1%, *P*=0.35) (Figure 2).

### SU11274 induces cytoreduction of erlotinib-resistant H1975 tumour xenograft *in vivo*

**Figure 3:**
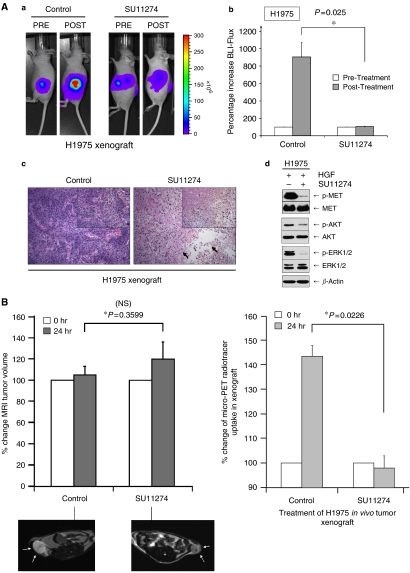

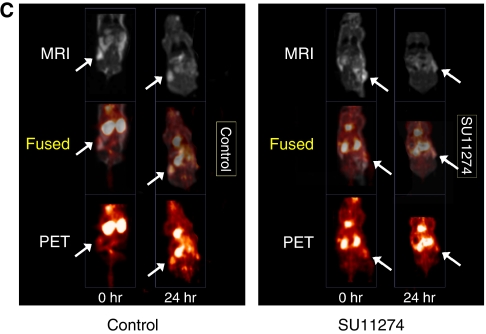
MET inhibition with SU11274 successfully induced *in vivo* tumour response in EGFR-TKI-resistant H1975 cells in murine xenograft model assessed by multimodal molecular imaging. (**A**) *In vivo* tumour xenografts for H1975-luc cells were established as described in the Materials and Methods section in 6-week old nude mice. Daily SU11274 (100 *μ*g per xenograft) treatment was administered to the H1975-luc lung cancer tumour xenografts in nude mice as described. DMSO diluent control was included for comparison. Imaging was performed using a Xenogen IVIS 200 System cooled CCD camera at indicated times. (a) Representative BLI digital pictures of nude mouse from each of the treatment conditions are illustrated. SU11274 significantly inhibited L858R/T790M-EGFR expressing H1975-luc *in vivo* tumorigenesis within the treatment durations (6 days). (b) Mean values of relative BLI flux of each group are plotted here (H1975-luc). *N*=4 per treatment group. Error bar, s.e.m. (^*^), *P*=0.025 for H1975-luc. Representative tumour xenograft micrographs from H1975-luc (c) cell line under haematoxylin and eosin (H&E) staining are also shown here for the control and SU11274 treatment animals. Magnification × 100 (inset, × 200). (d) SU11274 inhibited HGF-driven signalling activation in H1975 cells. H1975 cells were stimulated with HGF (50 ng ml^−1^, 15 min) and inhibited by MET inhibitor SU11274 (1 *μ*M, 4 h) *in vitro*, and analysed with 7.5% SDS–PAGE and immunoblotting with the indicated antibodies as described in Materials and Methods. (**B**, **C**) Magnetic resonance imaging (MRI) and microPET molecular imaging studies of MET inhibition of H1975 *in vivo* xenograft. H1975 *in vivo* xenografts were established as above for treatment with either diluent control (*N*=2) or SU11274 (*N*=2). The nude mice with H1975 xenografts were subjected to MRI and microPET imaging as described in the Materials and Methods section at 0, and 24 h with the MET inhibitor SU11274 treatment or diluent control. (**B**) Examples of the transverse sections of high-resolution MRI images of the tumour xenografts at baseline between the two treatment groups were shown here for illustration (left). The MRI tumour volumes were analysed digitally with the calculated tumour volume changes at the indicated time intervals (0 and 24 h) plotted. Comparing with baseline, the control xenograft tumour volume increased by 105.1±8.3% at 24 h, whereas the SU11274-treated xenografts increased by 120.2±16.2% at 24 h. The MRI tumour volumes changes at 24 h post-treatment between the two groups were not statistically significant (^*^P=0.360). Error bar, s.e.m. Quantitative microPET radiotracer uptake of the H1975 tumour xenografts at 60 min of radiotracer tail-vein infusion in the animals' pretreatment baseline (0 h) and post-MET-TKI treatment at 24 h is shown graphically (right). *N*=2 in each treatment group: Control and SU11274. Error bar, s.e.m. Representative co-registered pictures of the microPET/MRI (low-resolution) images of each xenograft from the two treatment groups are shown in (**C**). SU11274 induced early tumour metabolic response, as early as 24-h post-TKI treatment, with statistically significant inhibition of glucose metabolism as evident in the decrease in microPET uptake signal intensity by 45% (*P*=0.0226) in SU11274-treated xenografts, when compared with diluent control. The degree of increase in the glucose uptake in the H1975 tumour xenograft in diluent control is also consistent with the average rate of the xenograft growth (increase of 50.8% BLI flux per day) as reflected in the bioluminescence imaging.

To further test the role of MET inhibition in EGFR-TKI-resistant lung cancer *in vivo*, we developed stable luciferase-expressing H1975 lung cancer cells using lentivirus transduction. These cells were used in an *in vivo* xenograft model coupled with multimodal molecular imaging for non-invasive monitoring of xenograft growth and tumour response to TKI. Daily treatment with the MET inhibitor SU11274 caused statistically significant interval retardation of the xenograft tumour growth of H1975 cells with a ninefold reduction (*P*=0.0251) in the xenograft growth, when compared with the diluent control, during the treatment period (Figure 3A-a,b). At the end of treatment period, SU11274-treated H1975 xenograft tumour BLI flux remained essentially unchanged at 104% (*P*=0.0251), when compared to 905% as seen in the diluent control (Figure 3A-a,b). Histological analysis of the tumour xenografts harvested at the end of the experiment confirmed the presence of intense tumour necrosis in the SU11274-treated animals, but not in the diluent control (Figure 3A-c). Both EGFR and MET signal pathways are functional and ligand-sensitive in the erlotinib-resistant H1975 cells. HGF stimulated downstream signal path activation in AKT (survival) and the mitogen-activated protein kinase ERK1/2 (proliferation–differentiation), as surrogate markers for MET inhibition were both abrogated in the H1975 cells by SU11274 treatment *in vitro* (Figure 3A-d). Furthermore, we also showed that SU11274 inhibition effectively induced erlotinib-resistant A549 xenograft cytoreduction *in vivo* (Supplementary Figure 1).

### SU11274 inhibition induces early tumour response of the H1975 *in vivo* xenograft evident in microPET/MRI studies

We focused further on the H1975 cell line to investigate whether MET inhibition with SU11274 induced H1975 xenograft tumour response in terms of glucose metabolism as monitored by *in vivo* FDG-PET (glucose analogue [^18^F]fluoro-2-deoxy-D-glucose-positron emission tomography) studies with MRI co-registration. *In vivo* SU11274 inhibition induced a metabolic tumour response in H1975 xenografts within 24 h of the first treatment dose (Figure 3B and C). Although the changes of the calculated xenograft tumour volumes between the two treatment groups did not differ significantly (*P*>0.05) (Figure 3B), the SU11274-treated xenografts had statistically significant lower glucose metabolism by 45% (*P*=0.0226), when compared to diluent control (Figure 3B and C) (also see Supplementary Figure 2).

### MET–EGFR signalling cross-activation in lung cancer

**Figure 4:**
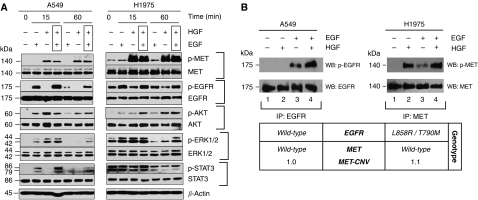
Signalling cross-activation between MET and EGFR signalling pathways. (**A**) Cross-activation between MET and EGFR signalling in lung cancer cells, A549 and H1975. A549 or H1975 cells were cultured in serum-starved conditions with exogenous stimulation with RTK ligands: EGF alone, HGF alone, or both EGF and HGF. Cells without any ligand stimulation were included as control. Both MET and EGFR signalling pathways are functional and ligand-sensitive in A549 and H1975 cells. There was augmented downstream signalling with combined EGF-HGF co-stimulation, with also more durable signalling induction. In A549 cells, although HGF alone did not activate EGFR phosphorylation appreciably, under co-stimulation conditions with EGF together, HGF further enhanced the EGFR phosphorylation in A549 cells to a level higher than that with EGF alone. On the other hand, EGF stimulation of H1975 cells co-activated MET receptor to enhance the level of MET phosphorylation. (**B**) MET–EGFR cross-activation in lung cancer. Left panel (A549), HGF cross-activated p-EGFR in A549 cells in the presence of co-stimulation with EGF. A549 cells were cultured in serum-starved conditions overnight, then stimulated with EGF alone (100 ng ml^−1^, 15 min), HGF alone (50 ng/ml, 15 min), or both. Whole cell lysates were collected for immunoprecipitation with EGFR antibody, followed by immunoblotting (WB) with antibodies against p-EGFR[Y1068] (upper panel) and total EGFR (lower panel). Right panel (H1975), EGF cross-activates phospho-MET in H1975 cells. H1975 cells were cultured in starved media overnight, then stimulated with EGF alone (100 ng ml^−1^, 15 min), HGF alone (50 ng ml^−1^, 15 min), or both. Whole cell lysates were collected for immunoprecipitation with MET antibody (C-12), followed by immunoblotting with antibodies against p-MET[Y1234/1235] (upper panel) and total MET (lower panel). The *MET* and *EGFR* genotypes of the A549 and H1975 cells, as well as their *MET* genomic copy numbers, are shown in the bottom.

As MET and EGFR often co-express in lung cancer cells (Figure 1), we asked if there is signalling cross-activation between MET and EGFR pathways. Both A549 (wild-type *EGFR*) and H1975 (L858R/T790M-*EGFR*) cell lines were used as models for the signalling studies (Figure 4). Here, MET and EGFR signal transduction pathways were both shown to be functional and ligand-sensitive, although H1975 cells have higher serum-independent constitutively activated MET and EGFR. Enhanced and more durable downstream signalling activation was observed in phospho-AKT (survival), phospho-ERK1/2 (proliferation-differentiation), and phospho-STAT3 (transcriptional activation) when A549 and H1975 cells were co-stimulated with dual-ligand (HGF and EGF) (Figure 4A; see lanes 4, 7, 11, and 14). Immunoprecipitation studies of the MET and EGFR under single- or dual-ligand stimulation confirmed the presence of receptor cross-activation between MET and EGFR in these lung cancer cell lines (Figure 4B). In A549 cells, HGF was capable of activating EGFR in the presence of EGF, whereas in H1975, EGF activated MET with and without co-stimulation with HGF.

### *MET* is activated with no genomic amplification or mutations in H1975 lung adenocarcinoma cells

Using standard QPCR technique, we determined the *MET* genomic copy number in several NSCLC cell lines, namely A549, H1975, H441, H520, H596, and H661. None of the cell lines examined exhibited *MET* genomic amplification. *MET* genomic copy number in H1975 was found to be 1.1, whereas that of A549 being 1.0 for comparison (Figure 4B). Direct DNA sequencing of the *MET* gene in H1975 did not reveal any non-synonymous mutations.

### Dual inhibition with SU11274 plus erlotinib/CL-387,785 potentiates the MET-targeted inhibitory efficacy in erlotinib-resistant lung cancer cells

**Figure 5:**
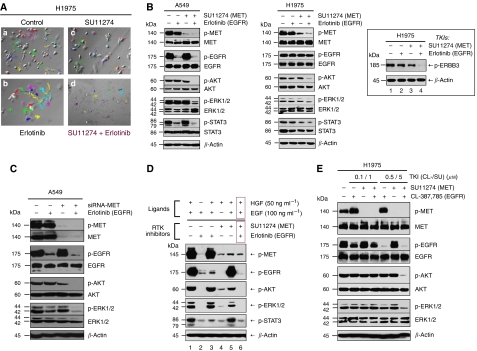
SU11274 inhibition of MET in combination with EGFR inhibitor in erlotinib-resistant NSCLC cell signalling. (**A**) Potentiated inhibition of cellular cytoskeletal functions by combined MET–EGFR inhibition (SU11274 plus erlotinib) in H1975 cells under video microscopy. H1975 cells had constitutively activated cytoskeletal functions with enhanced cell motility and migration under the serum-starved culture conditions. Comparing with the untreated control (left panel), drug treatment using SU11274 (right panel) substantially inhibited the constitutively activated cytoskeletal functions of H1975 cells (Supplementary Figure 3). H1975 cells were cultured in serum-stimulated conditions (10% FBS) and treated with the following for video microscopy digital video recording as described in the Materials and Methods: (a) DMSO diluent control, (b) Erlotinib alone (2 *μ*M), (c) SU11275 alone (5 *μ*M), and (d) combined concurrent SU11274 (5 *μ*M)+erlotinib (2 *μ*M). Complete abrogation of cytoskeletal functions with inhibition of cell motility and migration was only evident in the dual SU11274/erlotinib TKI-treated cells (d). (**B**) MET inhibition using SU11274, in combination with EGFR inhibition (erlotinib), induced cooperative downstream signalling inhibition in A549 (left panel) and H1975 (right panel) cells *in vitro*. EGFR-TKI-resistant A549 and H1975 cells were cultured in serum-starved conditions with EGF and HGF dual ligands stimulation as described in the Materials and Methods section. The cells were treated with SU11274 alone, erlotinib alone, or combination SU11274 plus erlotinib, then analysed in immunoblotting as indicated. (**C**) MET inhibition using specific siRNA-*MET*, in combination with EGFR inhibition (erlotinib) induced cooperative downstream signalling inhibition in A549 cells *in vitro*. Cells were transfected with control siRNA or siRNA-*MET* as described in Methods. Forty-eight hours after transfection, cells were cultured in starved media overnight, then treated with or without erlotinib and alone or in combination with siRNA-*MET* as indicated. After 4 h of inhibitor treatment, cells were then stimulated with both EGF (100 ng ml^−1^) and HGF (50 ng ml^−1^) ligands as indicated for 15 min. Whole cell lysates were then collected for immunoblotting analysis as in panel B above. (**D**) Rescue from alternative RTK ligand-stimulated signalling (MET–HGF *vs* EGFR–EGF) against TKI in A549 cells. A549 cells were cultured under serum-starved conditions, and then treated with either HGF or EGF, and in the presence or absence of the corresponding targeted inhibitor SU11274 or erlotinib as indicated (lanes 2, 4). Dual ligand stimulation (HGF and EGF) with single or dual TKIs treatment was included as indicated (lanes 1, 3, 5, 6). Although receptor-specific TKI was able to inhibit the downstream signalling driven by the corresponding ligand stimulation, alternative ligand stimulation in the form of dual ligand stimulation rescued the inhibited downstream signals. Dual TKI SU11274 plus erlotinib inhibition was required to fully knockdown the dual ligand-stimulated downstream signal activation of AKT, ERK1/2, and STAT3 (lane 6). (**E**) MET inhibition with SU11274, in combination with EGFR inhibition using CL-387,785 (irreversible EGFR-TKI), induced cooperative downstream signalling inhibition in H1975 cells *in vitro*. Cells were cultured in starved media overnight, then treated with or without CL-387,785 and alone or in combination with MET inhibitor SU11274 as indicated. After 4 h of inhibitor treatment, cells were then stimulated with both EGF (100 ng ml^−1^) and HGF (50 ng ml^−1^) ligands as indicated for 15 min. Whole cell lysates were then collected for immunoblotting analysis. Similar to erlotinib, CL-387,785 further sensitised H1975 cells to SU11274 inhibition with enhanced cooperative inhibition of signalling pathways downstream of the two RTKs.

As both MET and EGFR are functional in erlotinib-resistant cell lines such as A549 and H1975 cells, and there is signalling cross-activation between the two receptors (Figure 4), we next investigated if MET inhibition could be enhanced in combination with an EGFR inhibitor. The EGFR-TKI-resistant H1975 cells were tested against SU11274 inhibition, either alone or in combination with erlotinib. We determined the functional effect of combined MET–EGFR inhibition on the cytoskeletal functions of H1975 cells. Cell motility and migration are crucial cellular regulatory functions in tumour cell invasion and metastasis. Video microscopy studies showed that SU11274 inhibition substantially abrogated the constitutively activated cytoskeletal changes of the serum-starved H1975 cells (with activated p-MET and p-EGFR), as reflected in the cellular migrational trajectories and velocity (Supplementary Figure 3). Moreover, SU11274 alone had some inhibitory effect on cytoskeletal functions in H1975 cells under serum-stimulated (10% FBS) conditions, whereas concurrent dual TKI combinatorial inhibition using SU11274 plus erlotinib completely abrogated the cytoskeletal functions (Figure 5A).

Using dual-ligand concurrent stimulation (HGF and EGF) to activate both MET and EGFR in A549 and H1975 cells, single TKI alone was relatively ineffective in inhibiting the downstream signalling completely (Figure 5B). On the other hand, concurrent dual inhibition with MET–EGFR TKIs (SU11274 plus erlotinib) effectively induced cooperative and enhanced inhibition of the key downstream proliferative/survival and anti-apoptotic signal paths (phospho-AKT, phospho-ERK1/2, and phospho-STAT3) (Figure 5B). Most interestingly, despite the fact that p-EGFR[Y1068] was not significantly inhibited under the dual SU11274/erlotinib combinatorial treatment in H1975 cells, p-ERBB3[Y1289] activation was effectively abrogated only under this dual inhibitory strategy (Figure 5B).

The signalling experiment using *MET*-specific siRNA instead of SU11274 in A549 cells (Figure 5C) showed similar inhibition synergism using dual RTK inhibition with siRNA-*MET* and erlotinib. Using the A549 cell line as a model, we further demonstrated that alternative ligand-stimulated RTK signalling (MET–HGF and EGFR–EGF) indeed could rescue the downstream signalling activation from single targeted inhibitor (Figure 5D), supporting our hypothesis of the optimal efficacy of dual MET–EGFR inhibition especially in the *in vivo* setting where both RTK signal paths are functional and activated. Irreversible EGFR-TKIs (Kobayashi *et al*, 2005b), such as CL-387,785 or HKI-272 (Ruhe *et al*, 2007), have been shown to exhibit inhibitory efficacy against erlotinib-resistant T790M mutation. Here, in H1975 cells, results similar to erlotinib were obtained using the irreversible EGFR-TKI, CL-387,785, in combination with SU11274 (Figure 5E).

**Figure 6:**
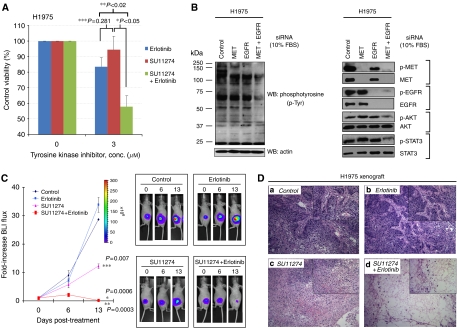
Dual SU11274–erlotinib inhibition induced cooperative inhibition in H1975 cell viability *in vitro* and murine xenograft tumour growth *in vivo*. (**A**) *In vitro* inhibition using MET inhibitor SU11274 combined with erlotinib was more effective in H1975 cell viability inhibition under serum-stimulated conditions. Enhanced inhibition of cell viability was evident with dual SU11274-erlotinib treatment in combination (at 3 *μ*M of each TKI as indicated). ^*^P<0.05 (SU11274/erlotinib *vs* SU11274); ^**^P<0.02 (SU11274/erlotinib *vs* erlotinib); and ^***^P=0.281 (SU11274 *vs* erlotinib). Error bar, s.d. (**B**) Combined knockdown of *MET* and *EGFR* signalling using short-interfering RNA (siRNA) in H1975 cells resulted in enhanced downstream signal transduction inhibition. H1975 cells cultured under serum-stimulated conditions were treated with siRNAs specifically targeted against mRNA of *MET* alone, *EGFR* alone, or both *MET* and *EGFR* as described in the Materials and Methods section. Cells with siRNA knockdown as indicated were harvested for immunoblotting using antibodies against phosphotyrosine (left panel) to survey the effects on global cellular phosphotyrosine phosphoproteomic profiles. Cells were also immunoblotted with antibodies against the MET and EGFR signal paths including the downstream pro-survival AKT and STAT3 pathways (right panel). Concurrent dual knockdown of *MET* and *EGFR* signalling by siRNA in H1975 cells led to optimally enhanced downregulation of global phosphorylated cellular proteome (left panel) including the pro-survival downstream p-AKT and p-STAT3 signal activation (right panel). (**C**) *In vivo* treatment using SU11274 combined with erlotinib induced cooperative complete regression of EGFR-TKI-resistant H1975 tumour xenograft growth. EGFR-TKI-resistant H1975-luc cells were used to establish nude mouse xenograft *in vivo* as described in the Materials and Methods section. The nude mice with H1975-luc xenografts were then treated with diluent control, EGFR inhibitor (erlotinib, 100 mg/kg/day) alone, MET inhibitor (SU11274, 50 *μ*g per xenograft per day) alone, or both inhibitors concurrently (SU11274 plus erlotinib). Tumour xenograft growth was monitored by BLI at pretreatment baseline (day 0), and on post-treatment days 6 and 13. SU11274, in combination with erlotinib, induced complete tumour xenograft regression of H1975 cells *in vivo*. The mean relative BLI flux from each treatment group was plotted graphically (*N*=4 per treatment group). Error bar, s.e.m. (^*^), (SU11274/erlotinib *vs* erlotinib) *P*=0.0006. (^**^), (SU11274/erlotinib *vs* SU11274) *P*=0.0003. (^***^), (SU11274 *vs* erlotinib) *P*=0.0070. (**D**) H1975 tumour xenograft micrographs under H&E staining at × 100 magnification (and × 200, inset) showed substantial viable tumour cells in panel (a) DMSO control, and (b) erlotinib-treated animals, whereas there were necrotic and apoptotic tumour cells seen in panel (c) SU11274 (suboptimal dose: 50 *μ*g per xenograft) and massively so in panel (d) in combined SU11274 plus erlotinib-treated animals.

As erlotinib is the only FDA-approved clinical EGFR-targeted inhibitor for the treatment of advanced lung cancer in the United States, we further investigated its potential use in combination with MET inhibition against erlotinib-resistant H1975 cells using an *in vitro* cellular viability assay and BLI tumour xenograft growth assay *in vivo*. Under serum-stimulated conditions, when both EGFR and MET were basally activated, SU11274 plus erlotinib (3 *μ*M of each TKI) induced a significantly enhanced cell viability inhibition (3 *μ*M: 58.0±6.8%, *P*<0.05), when compared with either erlotinib or SU11274 treatment alone (Figure 6A). To further validate this combination dual TKI strategy, we also subjected the H1975 cells to pathway-specific siRNA knockdown of the MET and EGFR kinase signal paths, either alone or in combination, under serum-stimulated conditions (Figure 6B). Optimal downstream signalling inhibition (phospho-AKT and phospho-STAT3) and global phosphotyrosine (p-Tyr) cellular signalling inhibition were achieved through dual inhibition with siRNA knockdown of both *MET* and *EGFR* targets, when compared with single target knockdown. Taken together, these data provide support that cooperative enhanced inhibition using dual TKIs against MET and EGFR pathways may be an effective treatment strategy to inhibit lung cancer with intrinsic or acquired T790M-EGFR-mediated TKI resistance.

Finally, we also tested concurrent dual SU11274 plus erlotinib combinatorial treatment in our *in vivo* H1975-luc BLI xenograft growth assay (Figure 6C). Here, a suboptimal daily dose of SU11274 (50 *μ*g per xenograft per day) was found to be partially effective in retarding the xenograft growth, when compared with either diluent control or erlotinib (100 mg kg^−1^ day^−1^) alone. Moreover, we found that combining SU11274 with erlotinib induced a complete tumour xenograft regression (0.12-fold BLI from baseline), evident within 2 weeks of dual inhibitor therapy. The difference seen with dual MET–EGFR-TKIs treatment is statistically significant when compared with either erlotinib alone (35.8-fold BLI increase, *P*=0.0006) or with SU11274 alone (12.2-fold, *P*=0.0003). These molecular imaging data were confirmed by xenograft H&E-stained histology examination (Figure 6D). In particular, the combined SU11274 plus erlotinib treatment resulted in substantial tumour necrosis.

## Discussion

At least half of the acquired resistance to EGFR-TKI in advanced NSCLC patients is thought to be mediated by the ‘gatekeeper’ mutation T790M in exon 20 of *EGFR* (Kobayashi *et al*, 2005a; Pao *et al*, 2005). Many tumours with intrinsic resistance to erlotinib/gefitinib were found to have wild-type *EGFR* and/or mutant *KRAS*. At present, no FDA-approved inhibitor drugs have been shown to be successful in overcoming T790M-mediated resistance clinically. Recent study suggested that T790M-EGFR-mediated resistance could even emerge from the irreversible EGFR/ERBB2 inhibitor HKI-272 treatment at maximally tolerated dosing, as it mediates resistance to low concentrations of the irreversible inhibitor (Godin-Heymann *et al*, 2008). Alternative novel therapies to target lung cancer patients with intrinsic or acquired resistance to erlotinib are needed. MET has recently been affirmed to be an attractive anti-neoplastic therapeutic target (Corso *et al*, 2005; Peruzzi and Bottaro, 2006; Salgia, 2006), including lung cancer (Christensen *et al*, 2003, 2005; Ma *et al*, 2003a, 2003b, 2005a, 2005b; Sattler *et al*, 2003). MET was found overexpressed in up to 67% of lung adenocarcinomas in our previous study (Ma *et al*, 2005a). Various targeted inhibitory strategies are being undertaken in drug development to antagonise MET/HGF signalling in human cancers, including small-molecule kinase inhibitors, antibodies to the ligand HGF, and receptor MET itself (Christensen *et al*, 2005; Martens *et al*, 2006; Peruzzi and Bottaro, 2006). In this study, we identified that there is frequent co-expression of MET and EGFR in NSCLC cell lines. In the erlotinib-resistant H1975 cell line (L858R/T7980M-*EGFR*), *MET* is neither genomically amplified nor mutated. Yet, MET is activated in the cells, possessing both constitutive (serum/ligand-independent) and basal (serum-stimulated) receptor activation. Furthermore, MET also remains HGF ligand-sensitive. Owing to the unique intrinsic properties of MET regulating cellular ‘invasive signalling’, MET has been proposed as not merely playing a role in ‘oncogene addiction’ in a small subset of human cancers but can also play an essential role in ‘oncogene expedience’ by inducing an enhanced transformed tumour malignant ‘fitness’ in a much larger range of cancers leading to promotion of tumour progression (Comoglio *et al*, 2008). And in the latter case, activated MET can intercept with various other oncogenic signals, including mutant-EGFR, in maintaining and enhancing the tumour invasive–progressive phenotype, thereby also allowing the opportunity for MET to be a therapeutic target even in late advanced metastatic disease. The MET inhibitor SU11274 was shown to promote apoptosis in H1975 cells, but was ineffective in the MET-negative/EGFR-negative H520 cells. SU11274 was previously characterised to be a selective, reversible ATP-competitive inhibitor of MET kinase (Sattler *et al*, 2003; Ma *et al*, 2005a). Here, we show that SU11274 exhibited inhibitory efficacy in the EGFR-TKI-resistant H1975 cells both *in vitro* and *in vivo*. In particular, it inhibited MET signalling, induced cellular apoptosis, and abrogated cytoskeletal functions (key controlling step in tumour invasion and metastasis) *in vitro* (Birchmeier *et al*, 2003), and was effective *in vivo*, leading to cytoreduction of murine tumour xenografts of the T790M-EGFR expressing erlotinib-resistant H1975 cells. Taken together, our study supports the hypothesis that MET may be targeted to circumvent T790M-EGFR-mediated intrinsic or acquired resistance to EGFR-TKI (erlotinib) in lung cancer.

H1975 cells were tested further to provide a better understanding of the mechanism of dual MET–EGFR inhibition. A549 cell line was included as model in signalling studies for comparison with H1975 cells. A549 is an extensively studied lung cancer cell line and is known to have *KRAS* mutation but not any *EGFR* kinase domain mutations. In our *in vitro* signalling studies, we identified that there was signalling cross-activation between MET and EGFR in both A549 and H1975 cells. Nonetheless, the pattern of cross-activation appeared to be different between the two cell lines. In A549 cells, HGF brought about cooperative induction of p-EGFR[Y1068] in the presence of EGF, whereas in H1975 cells, EGF induced cross-activation of p-MET[Y1234/1235] by itself and further MET activation when combined with HGF in dual ligand stimulation. It is tempting to postulate that the different pattern of cross-activation observed in A549 and H1975 cells might be a result of the different *EGFR* kinase mutational status in the two cell lines, that is non-mutated in A549 but L858R/T790M in H1975. The mutant EGFR in H1975 evidently is capable of cross-activating MET in an EGF –ligand-dependent manner, indicating that MET could be ‘downstream’ of the mutant EGFR in H1975. Of interest, it has recently been shown that the MET receptor activating phosphorylation site was highly responsive to EGFRvIII levels in glioblastoma cells *in vitro*, suggesting downstream cross-activation of MET by mutant EGFRvIII (Huang *et al*, 2007).

L858R/T790M-*EGFR* mutations exist in H1975 cells in-*cis* (Pao *et al*, 2005). The double mutations not only confer resistance to gefitinib/erlotinib but also result in markedly enhanced catalytic kinase and oncogenic activity (Mulloy *et al*, 2007). Emerging evidence suggests that the T790M ‘gatekeeper’ mutation may exist in lung tumours before EGFR-TKI therapeutic selection (Bell *et al*, 2005; Godin-Heymann *et al*, 2007), partly due to its enhanced oncogenicity, and accounts for the adverse clinical course and outcome in gefitinib/erlotinib-resistance lung cancers after a course of rapid TKI selection. Interestingly, we found that H1975 cells co-express EGFR and MET at high level, although without any *MET* genomic amplification, and were capable of serum-independent constitutive MET activation. Moreover, MET and EGFR engage in collaborative signalling cross-activation to transduce stronger and more durable downstream signals. A recent report utilised a mutant *EGFR* (deletion 19)-expressing NSCLC cell line HCC827, which is highly sensitive to gefitinib, an *in vitro* gefitinib-resistance long-term inhibition culture system to select for gefitinib-resistant cell subclones (Engelman *et al*, 2007). Acquired *MET* amplification was identified to be a potential alternative mechanism to enable the mutant *EGFR*-expressing HCC827 to become secondarily resistant (HCC827-GR) without a T790M mutation. In the course of our study, Bean *et al* (2007) reported the presence of *MET* amplification that occurred independently with and without T790M-*EGFR* mutation in lung tumours. *MET* amplification in gastric cancer cell lines has recently been correlated with high sensitivity towards MET inhibitor (Smolen *et al*, 2006). Interestingly, another recent report adopted a global phosphoproteomic approach using cell lines sensitive to gefitinib (HCC827) and sensitive to SU11274 (MKN45), and showed that besides p-EGFR inhibition, gefitinib also inhibited p-MET (that is indeed constitutively activated) in HCC827, but not vice versa (Guo *et al*, 2008). In the case of SU11274, the MET inhibitor inhibited p-EGFR in MKN45 besides p-MET, but not vice versa (Guo *et al*, 2008). Further studies to examine the signalling cross-talk between the EGFR and MET receptor pathways in the context of mutations and downstream signalling networking would be helpful in optimising combinational therapeutic strategies. Moreover, it would be useful to further investigate and catalogue the activating mechanisms of *MET* (such as activating mutations, transcriptional and protein overexpression), and their role in oncogenic signalling in the context of multiple receptor co-activation in lung cancer.

Our report here demonstrates the efficacy of dual RTK targeted inhibition against MET (SU11274) and EGFR (erlotinib or CL-387,785) as a strategy to achieve optimised inhibition of cytoskeletal functions, cell viability, cellular signalling, and *in vivo* xenograft complete regression in T790M-EGFR-mediated erlotinib resistance. The concentrations of SU11274 used in our current study are consistent with previous results to be within the range of selectivity towards MET (Sattler *et al*, 2003; Ma *et al*, 2005a). Finally, our dual siRNA knockdown experiment against *MET* and *EGFR* (Figure 6B) in H1975 cells provides further validation of this novel therapeutic approach. Dual inhibition may be of benefit over single target inhibition, especially in the context of serum and/or alternative ligand stimulation. This can be of clinical relevance considering that tumour cells often exist and adapt *in vivo* under a multitude of host stromal conditions during various stages of tumour progression, including serum starvation (e.g. in tumour core), serum-stimulation, and also potentially microenvironment-specific ligand(s)-stimulation.

Dual TKI combinatorial approach may allow more effective target inhibition with a lower MET inhibitor concentration requirement, when compared with monotherapy alone, as suggested in our *in vivo* H1975-luc xenograft study. The ability to use lower drug concentrations than that in monotherapy would be beneficial and clinically relevant to minimise additive toxicity profiles of two inhibitor drugs of similar class used in combination. It is intriguing that we consistently observed a modest, but readily detectable degree of potentiated inhibition of MET phosphorylation by erlotinib, with and without SU11274, both in A549 and in H1975 cells. In addition, our siRNA-*EGFR* knockdown study in H1975 cells resulted in appreciable downregulation of p-MET[Y1234/1235], suggesting that the mutant EGFR in H1975 cells might signal into MET as a ‘downstream’ cross-talk collaborative signal partner (Figure 6B). It may account for some of the enhanced inhibitory effects seen in the dual TKI treatment. In our *in vivo* bioluminescence xenograft model, combined SU11274–erlotinib inhibition remarkably induced complete H1975-luc tumour xenograft regression associated with histological features of massive tumour necrosis–apoptosis. We were initially intrigued that despite the lack of effective inhibition of EGFR phosphorylation (at pY1068) by erlotinib alone, the drug enhanced inhibitory efficacy both *in vitro* and *in vivo* in H1975 cells when combined with SU11274. Engelman *et al* (2007) recently reported that *MET*, when amplified genomically as in the setting of acquired EGFR-TKI resistance, can capture the ERBB3 signal control from EGFR. Our data here suggest that MET inhibition (SU11274) in H1975 cells had a modest but detectible negative effect on ERBB3 activation, and that dual SU11274/erlotinib inhibition cooperatively abrogated p-ERBB3 signal activation completely (Figure 5B). This might partially explain the observed role of erlotinib in the dual inhibitory strategy against the T790M-*EGFR* mutant cells, even though it is ineffective against p-EGFR itself. Further studies to dissect the interplay between MET and ERBB3 signal paths in lung cancer are warranted. Besides the possible effect of erlotinib upon MET activation *per se*, one might not rule out other potential off-target effects of erlotinib as contributing factors. Stegmaier *et al* (2005) first reported a previously unrecognised EGFR-independent mechanism of gefitinib in inducing the differentiation and inhibiting proliferation of EGFR-negative acute myeloid leukaemia cells at clinically achievable doses (Stegmaier *et al*, 2005). More recently, erlotinib was also found to exhibit off-target anti-neoplastic effects in acute myeloid leukaemia and myelodysplastic syndrome, supporting the potential clinical therapeutic utility of these EGFR-TKIs in haematologic malignancies (Boehrer *et al*, 2007). As erlotinib is currently the only clinically approved EGFR-TKI for lung cancer in the United States, discovery of its utility in combinatorial inhibitory approaches would be of potential clinical benefit. Combination strategy to target EGFR and MET has recently been reported to show promise in overcoming mutant-EGFRvIII-driven glioblastoma, although using a higher concentration range of MET inhibitor (Huang *et al*, 2007). Some success to overcome T790M-mutant EGFR resistance has been reported using irreversible EGFR/ERBB family inhibitors, such as CL-387,785 and HKI-272 (Kobayashi *et al*, 2005b; Wong, 2007). Nonetheless, recent work in a L858R/T790M-*EGFR* transgenic mouse model suggests that the double mutant-*EGFR* responds only partially to HKI-272 alone (Li *et al*, 2007), and enhanced inhibition was seen in combination with mammalian target of rapamycin inhibitor.

In conclusion, the present study identified that despite having no genomic *MET* alterations in H1975 cells, the MET–HGF signal path is functional and activated in this EGFR-TKI-resistant cell line that already expresses the oncogenic mutant EGFR (L858R/T790M) signal axis. There are also receptor cross-activation and signalling circuitry cross-talk between MET and EGFR, and MET inhibition has efficacy *in vitro* and *in vivo* in the erlotinib-resistant H1975 cells. Our results also implicate that combination treatment using a MET inhibitor plus a reversible or irreversible EGFR kinase inhibitor to achieve dual MET–EGFR inhibition may represent an alternative strategy to circumvent T790M-*EGFR*-mediated resistance in lung cancer. Importantly, erlotinib may still have clinical utility in this context of combined inhibition with MET inhibitor in EGFR-TKI-resistant lung cancer. As T790M-EGFR may play a role in both intrinsic and acquired EGFR-TKI resistance in lung cancer, it would be useful to test concurrent combinatorial MET–EGFR inhibitors in clinical trials on lung cancer patients refractory to erlotinib/gefitinib. Other emerging pan-ERBB class inhibitors, such as PF00299804, or EGFR-targeting dual/multitargeted inhibitors, such as lapatinib (EGFR/ERBB2 inhibitor) or ZD6474 (EGFR/VEGFR2 inhibitor), might also be candidates for combination with MET inhibitors. Whether a combined MET–EGFR inhibitory strategy as upfront treatment is superior to MET inhibition used only after EGFR-TKI monotherapy failure should be the subject of further investigation.
