# Supplementary figures and images for: Dual MET–EGFR combinatorial inhibition against T790M-EGFR-mediated erlotinib-resistant lung cancer
Source: Br J Cancer. 2008 Aug 26;99(6):911–22. doi: 10.1038/sj.bjc.6604559 (PMC2538758; doi:10.1038/sj.bjc.6604559)

## Slide 1
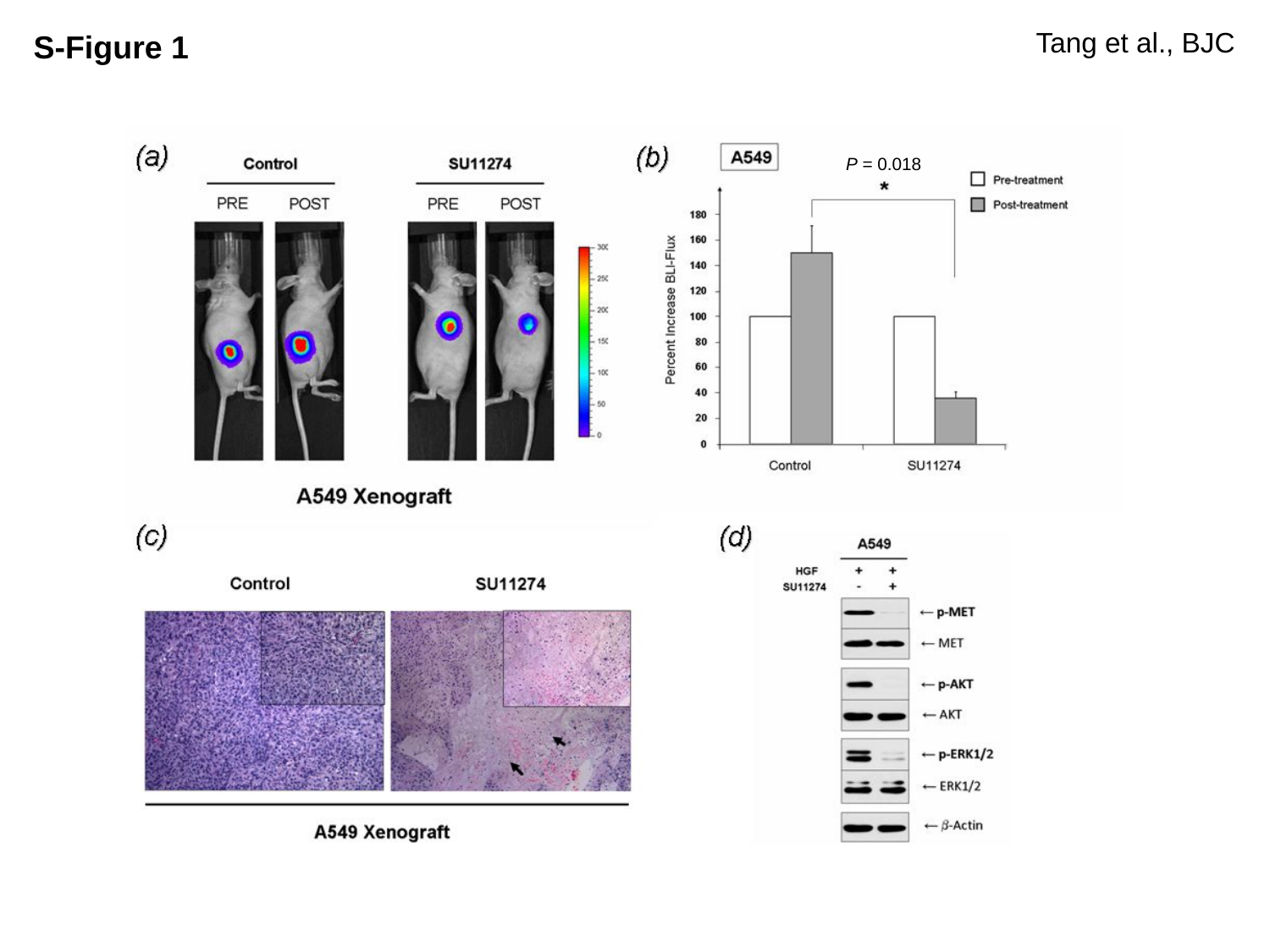

Tang et al., BJC
S-Figure 1
P = 0.018

## Slide 2
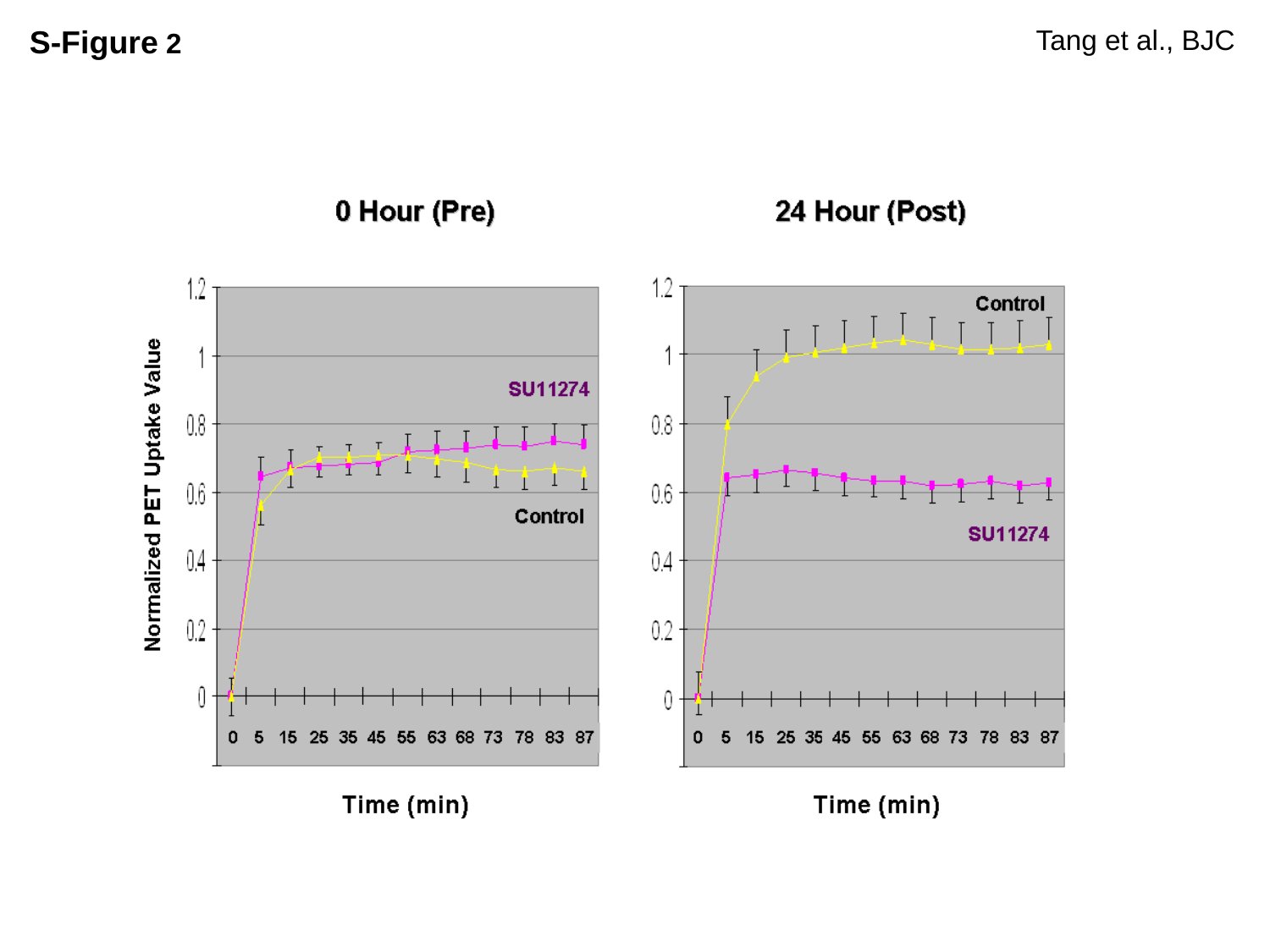

S-Figure 2
Tang et al., BJC

## Slide 3
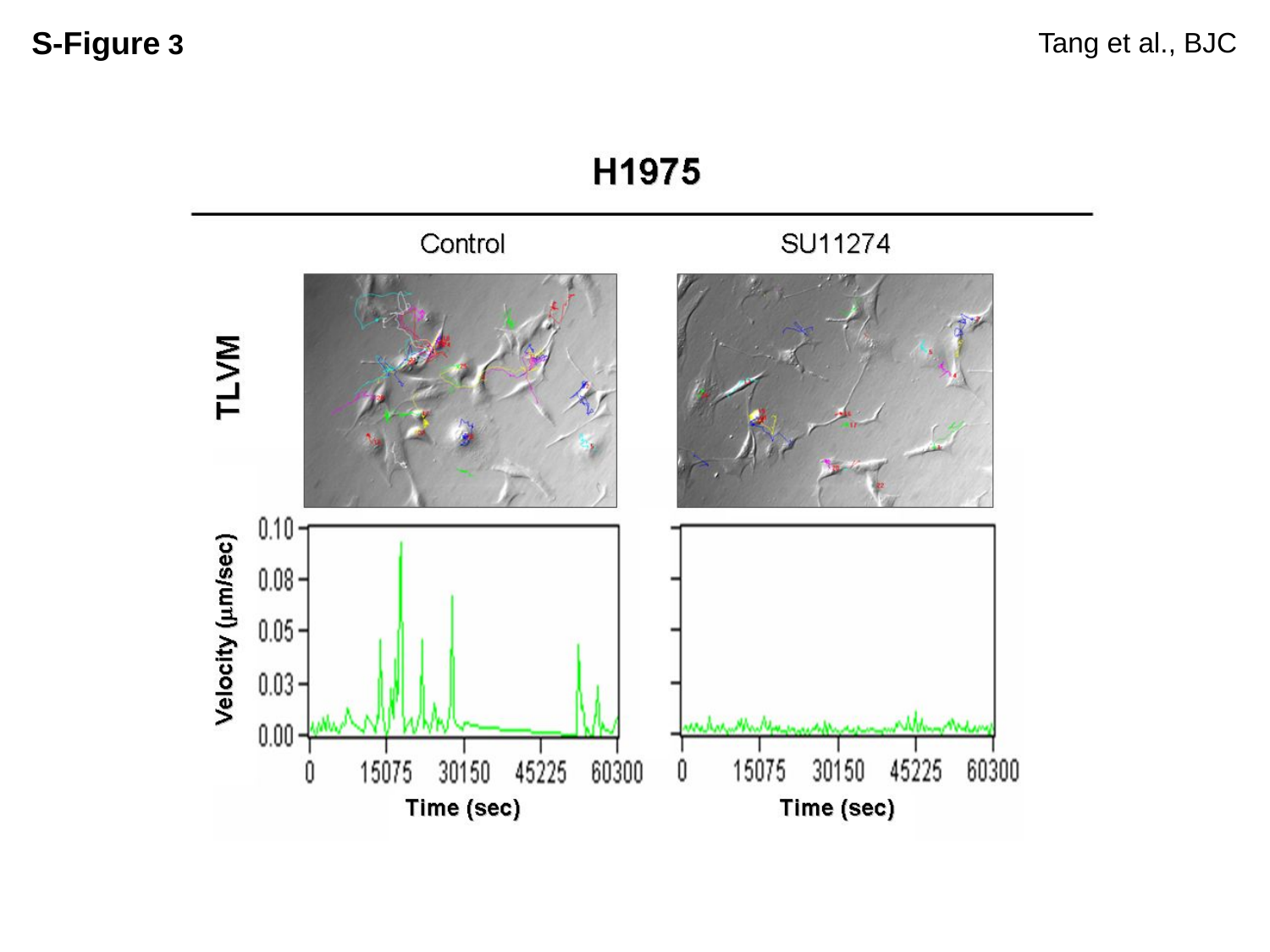

S-Figure 3
Tang et al., BJC

Supplement: Supplementary Figures [file 6604559x1.ppt]
