# Supplementary material for: Dual MET–EGFR combinatorial inhibition against T790M-EGFR-mediated erlotinib-resistant lung cancer
Source: Br J Cancer. 2008 Aug 26;99(6):911–22. doi: 10.1038/sj.bjc.6604559 (PMC2538758; doi:10.1038/sj.bjc.6604559)
Supplement: Supplementary Table [file 6604559x2.ppt]

## Slide 1
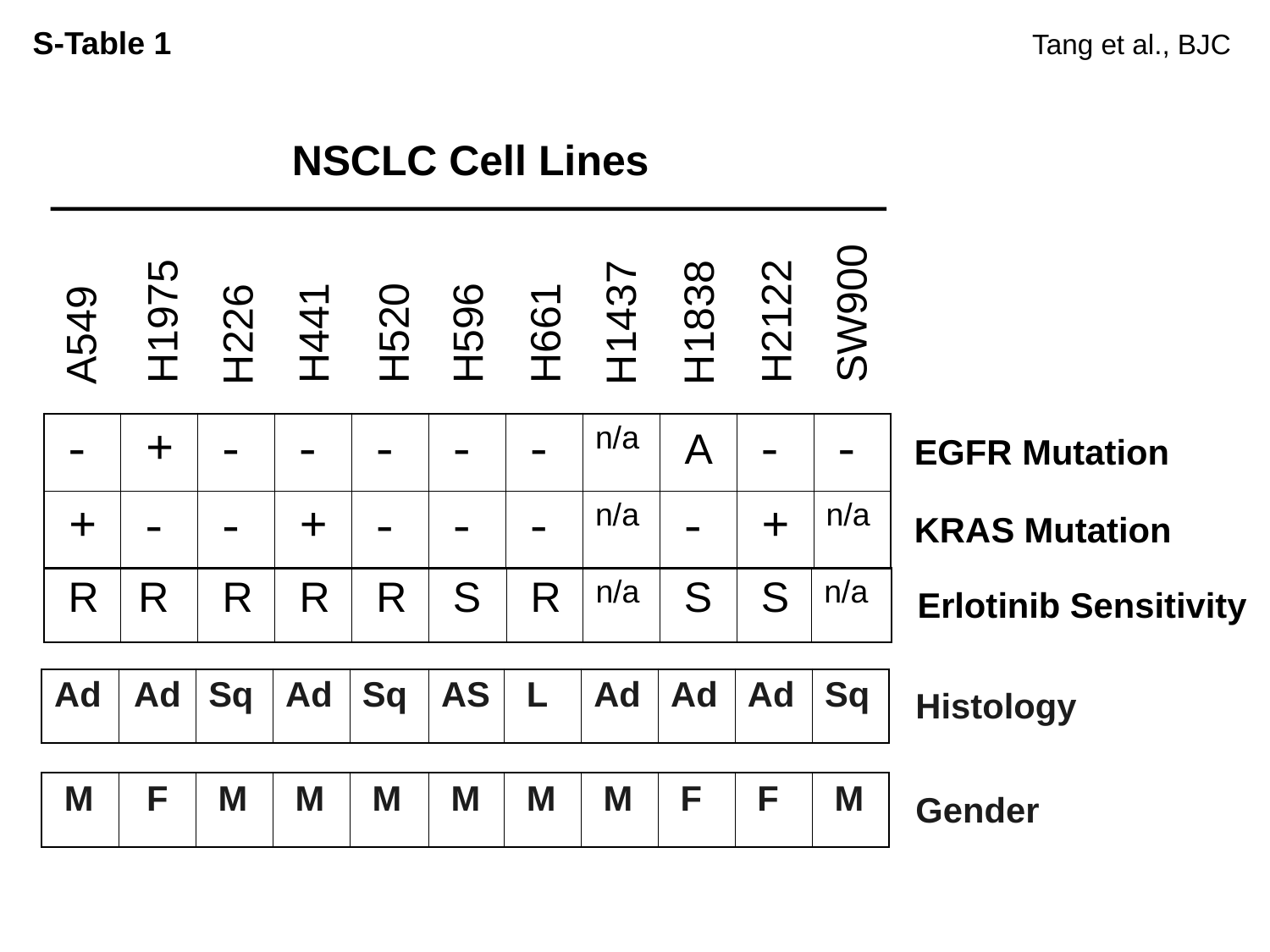

S-Table 1
Tang et al., BJC
NSCLC Cell Lines
SW900
H2122
H1975
H1437
H1838
H441
H520
H596
H661
H226
A549
| - | + | - | - | - | - | - | n/a | A | - | - |
| --- | --- | --- | --- | --- | --- | --- | --- | --- | --- | --- |
| + | - | - | + | - | - | - | n/a | - | + | n/a |
EGFR Mutation
KRAS Mutation
| R | R | R | R | R | S | R | n/a | S | S | n/a |
| --- | --- | --- | --- | --- | --- | --- | --- | --- | --- | --- |
Erlotinib Sensitivity
| Ad | Ad | Sq | Ad | Sq | AS | L | Ad | Ad | Ad | Sq |
| --- | --- | --- | --- | --- | --- | --- | --- | --- | --- | --- |
Histology
| M | F | M | M | M | M | M | M | F | F | M |
| --- | --- | --- | --- | --- | --- | --- | --- | --- | --- | --- |
Gender
